# TMS–EEG signatures of motor network dysfunction in multiple sclerosis

**DOI:** 10.1093/braincomms/fcag028

**Published:** 2026-01-31

**Authors:** Giorgio Leodori, Marco Mancuso, Davide Maccarrone, Matteo Tartaglia, Maria Ilenia De Bartolo, Angelo Collura, Stefano Pellegrini, Leonardo Malimpensa, Daniele Belvisi, Gina Ferrazzano, Ulf Ziemann, Antonella Conte

**Affiliations:** Department of Human Neurosciences, Sapienza University of Rome, Rome 00189, Italy; IRCCS Neuromed, Pozzilli 86077, Italy; Department of Human Neurosciences, Sapienza University of Rome, Rome 00189, Italy; Department of Human Neurosciences, Sapienza University of Rome, Rome 00189, Italy; Department of Human Neurosciences, Sapienza University of Rome, Rome 00189, Italy; Department of Human Neurosciences, Sapienza University of Rome, Rome 00189, Italy; IRCCS Neuromed, Pozzilli 86077, Italy; Department of Human Neurosciences, Sapienza University of Rome, Rome 00189, Italy; Department of Human Neurosciences, Sapienza University of Rome, Rome 00189, Italy; Department of Human Neurosciences, Sapienza University of Rome, Rome 00189, Italy; Department of Human Neurosciences, Sapienza University of Rome, Rome 00189, Italy; IRCCS Neuromed, Pozzilli 86077, Italy; Department of Human Neurosciences, Sapienza University of Rome, Rome 00189, Italy; Department of Neurology & Stroke, University Hospital Tübingen, Tübingen 72076, Germany; Hertie-Institute for Clinical Brain Research, University Hospital Tübingen, Tübingen 72076, Germany; Department of Human Neurosciences, Sapienza University of Rome, Rome 00189, Italy; IRCCS Neuromed, Pozzilli 86077, Italy

**Keywords:** transcranial-evoked potentials, brain oscillations, biomarkers, disease activity, longitudinal assessment

## Abstract

Multiple sclerosis (MS) progressively impairs brain network function, often driving disability even in the absence of overt structural MRI changes. Current clinical and radiological tools frequently fail to capture early, subtle disruptions in cortical activity that may indicate ongoing disease progression. Functional assessment methods capable of detecting these early network alterations are therefore critically needed. This study aimed to determine whether brain responses recorded by combining transcranial magnetic stimulation (TMS) with electroencephalography (EEG) from the primary motor cortex differ in MS, correlate with clinical disability and predict disease activity. Sixty-nine right-handed participants [mean age: MS 38.5 ± 9.1 years, healthy controls (HCs) 36.9 ± 8.8 years; 41 females] were enrolled, including 43 patients with relapsing–remitting MS and 26 HCs matched for age and sex. MS patients were clinically stable and off corticosteroids or CNS-acting medications at least 1 month prior to testing. All underwent single-pulse stimulation over the left primary motor cortex during EEG recording. Transcranial-evoked potentials (TEPs) and spectral perturbations were extracted. Patients were followed for 2 years and classified as active or stable based on ‘No Evidence of Disease Activity-3’ criteria. Patients showed significantly reduced P60 amplitude compared with controls (*P* = 0.0098, FDR-corrected *P* adj. = 0.0491), and a trend-level reduction in gamma-band desynchronization (i.e. less negative values) (*P* = 0.025, *P* adj. = 0.075), which correlated inversely with 9-Hole Peg Test times (*r*_s_ = −0.504, *P* = 0.001). A trend towards lower P15 amplitude was observed in patients with active disease (*P* = 0.0178, *P* adj. = 0.0891), and P15 amplitude significantly predicted disease stability at 2 years (accuracy = 74.4%, *P* = 0.023). TMS combined with EEG detects altered motor cortical network dynamics in MS. Less-pronounced (i.e. less negative) gamma-band desynchronization correlated with preserved fine-motor network efficiency, potentially reflecting a compensatory mechanism. The P15-evoked potential amplitude may predict disease activity. This perturbation-based approach provides a privileged window into network dysfunction in MS, with potential to guide early prognosis and treatment.

## Teaser

Leodori *et al*. report that transcranial magnetic stimulation combined with electroencephalography (TMS–EEG) captures differences in cortical motor network responses in multiple sclerosis. TMS–EEG measures correlate with manual dexterity and provide modest prediction of 2-year disease stability, highlighting their potential as functional biomarkers.

## Graphical Abstract

**Graphical Abstract:**
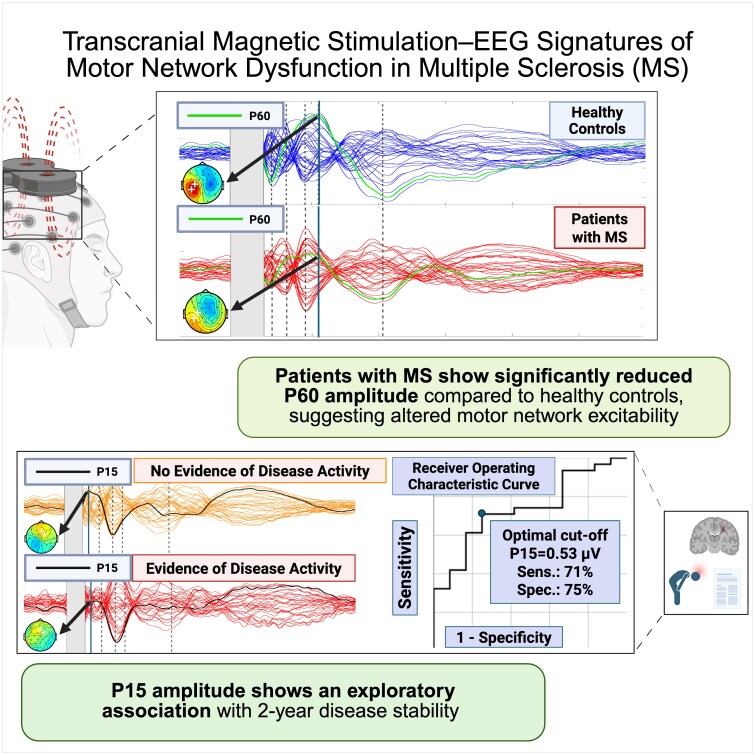

## Introduction

Multiple sclerosis (MS) is a chronic inflammatory and neurodegenerative disease of the central nervous system (CNS), characterized by demyelination, axonal loss and synaptic dysfunction.^1^ Although MS is initially dominated by inflammatory relapses, long-term disability often driven by insidious neurodegeneration and early functional network disruption.^2,3^ Although disease-modifying therapies (DMTs) reduce relapse rates and radiological activity, they often fail to prevent smouldering disease progression, which remains difficult to detect at an early stage—precisely when intervention might be most effective. Recent evidence has highlighted the critical role of synaptic dysfunction and brain network disintegration in MS progression, even in the absence of overt structural MRI changes.^4-7^ As a result, functional assessment tools capable of detecting early alterations in cortical activity are of great interest.

Motor disability represents a central contributor to long-term functional impairment in MS and motor network efficiency explains a large amount of variability in EDSS scores.^8^ The primary motor cortex (M1), as the origin of the corticospinal tract and a central hub in the motor network, is particularly relevant for studying mechanisms underlying motor impairment. Historically, transcranial magnetic stimulation (TMS) over M1 combined with EMG recording of motor-evoked potentials (MEPs) has been widely used to demonstrate diagnostic abnormalities in corticospinal conduction, as well as pathophysiological alterations in cortical excitability, synaptic plasticity and transcallosal motor transmission in MS.^9-11^ Most recently, the combined use of TMS and electroencephalography (TMS–EEG) enabled non-invasive interrogation of excitability and connectivity of the stimulated region with millisecond resolution.^12^ TMS-evoked potentials (TEPs) represent time-locked EEG responses to TMS and provide a window onto both the local reactivity of the targeted cortex and the dynamic interactions of the distributed networks to which it belongs.^13-15^ Accordingly, TEPs elicited from M1 are readouts of network-level excitability rather than mere local cortical integrity,^13,16,17^ a perspective that is particularly pertinent for MS, a disorder of distributed brain networks.^4-7^ In M1 TMS–EEG, components within ∼50 ms are thought to reflect reverberant activation of local cortical circuits, indexing local excitability and gain control.^14,15^ Components beyond ∼50 ms likely capture a mixture of continued local reverberation via cortico-cortical and subcortico-cortical loops together with propagation to remote nodes of the sensorimotor network to which M1 belongs;^18,19^ however, these later responses are more susceptible to sensory co-stimulation confounds.^20,21^ Preliminary evidence suggests that TEPs elicited from M1 may be altered in MS patients even at early disease stages, before disability occurs,^22^ and could be sensitive to pathophysiological mechanisms underlying typical symptoms such as motor fatigue.^23,24^ However, current evidence on TEP alterations in MS remains preliminary, with findings often limited to relatively late components whose specificity for local cortical excitability is uncertain.^25^ Further studies are needed to confirm these alterations, clarify their neurophysiological significance and determine whether they hold predictive value for long-term disease progression.

In parallel, time–frequency analysis (TFA) of TEPs can quantify TMS-related spectral perturbations (TRSPs), reflecting oscillatory responses triggered by external cortical stimulation across various frequency bands.^26,27^ TRSPs represent transient modulations in ongoing brain oscillations, closely related to endogenous, behaviourally relevant neural rhythms.^28-30^ Given the critical role of specific oscillatory neural activities (particularly mu- and beta-band rhythms) in sensorimotor function in MS,^31-33^ detecting changes in TRSP from M1 in MS can yield valuable pathophysiological insights into motor impairment. Importantly, if linked to specific clinical features, these spectral perturbations have the potential to serve as surrogate biomarkers to guide novel therapeutic strategies, including EEG-informed non-invasive brain stimulation.^34,35^ Combining TEP and TFA measures may thus offer a more comprehensive understanding of motor network integrity in MS than has hitherto been obtained.

This study aimed to assess whether TEP and TFA features elicited by single-pulse TMS of M1 could differentiate MS patients from healthy controls (HCs), relate to clinical disability and predict longitudinal disease stability. Specifically, we tested to what extent (i) MS patients show altered TEP amplitudes, latencies and TRSPs compared with HCs; (ii) these features correlate with disease duration, clinical measures of global disability and motor performance; and (iii) baseline TEP or TRSP metrics predict 2-year disease status defined by the No Evidence of Disease Activity (NEDA-3).^36,37^

## Materials and methods

### Subjects

Sixty-nine participants were included in the study: 43 patients with relapsing–remitting MS, diagnosed according to the 2017 revised McDonald criteria,^38^ and 26 HCs. The study was approved by the local ethics committee and conducted in accordance with the principles of the Declaration of Helsinki. All participants provided written informed consent prior to participation. Inclusion criteria for all participants were: no history of neurological or psychiatric illness (other than MS for patients), right-handedness, and no use of medications acting on the CNS such as antispasticity agents, anticonvulsants, antidepressants and anxiolytics (other than DMT). Additional inclusion criteria for MS patients were: clinical stability for at least 1 month, no corticosteroid treatment or changes in DMT in the month preceding the study. MS patients underwent baseline (T0) clinical evaluation, including the Expanded Disability Status Scale (EDSS), the assessment of manual dexterity with the right-hand 9-Hole Peg Test (9HPT) and experimental TMS–EEG recording. Patients were then followed longitudinally for 2 years with ∼6-monthly visits (earlier if new symptoms), including neurological examination with EDSS, documentation of relapses and brain MRI (assessment of new/enlarging T2 lesions and gadolinium-enhancing lesions). Based on this follow-up, they were classified after 2 years (T1) as either NEDA (no evidence of disease activity) or EDA (evidence of disease activity) according to the NEDA-3 criteria,^36,37,39^ which include absence of clinical relapses, no new or enlarging T2 lesions or gadolinium-enhancing lesions on MRI, and no confirmed disability progression as measured by the EDSS.

### TMS–EEG acquisition

A total of 120 single-pulse TMS pulses were delivered over the left M1 at 90% of each participant’s resting motor threshold (RMT), using a figure-of-eight coil (70 mm diameter) connected to a biphasic stimulator (Magstim Rapid). The interval between pulses was randomly jittered between 1100 and 1400 ms. The coil was positioned tangentially to the scalp at the hotspot for the right first dorsal interosseous (FDI) muscle, with the handle oriented ∼45° from the midline in a posterior–anterior direction. In this orientation, the biphasic pulse generated a posterior-to-anterior first half-cycle current in the cortex, followed by an anterior-to-posterior half-cycle. Coil position was monitored throughout the experiment using a neuronavigation system (SofTaxic, EMS) with an optical tracking system (Polaris Vicra, Northern Digital Inc., Canada). A subject-specific MRI surrogate was generated for each participant by scaling an MNI template head model to that participant’s cranial landmarks (nasion, left/right preauricular points, inion) and was used for neuronavigation. EMG was recorded from the right FDI using bipolar Ag/AgCl surface electrodes with a hand-dorsum ground, band-pass filtered (10–1000 Hz), amplified ×1000 (Digitimer D360), sampled at 5 kHz (CED 1401) and stored for offline analysis. Right-FDI EMG was also monitored online; trials showing pre-TMS activation (>0.02 mV within 1 s before TMS) were immediately discarded (≤2–3 per participant) and not stored for offline analysis (Signal Software V 6.0, CED). EEG was recorded with a 32-channel cap (BrainCap, EASYCAP) with electrodes positioned according to the international 10–20 system, using a DC-coupled amplifier (NeurOne, Bittium) and a sampling rate of 10 kHz. An additional electrode at Fpz served as ground and POz as online reference. Impedance at all electrodes was maintained below 5 kΩ. During TMS, participants wore noise-reduction earmuffs and earphones continuously playing a noise matched to the spectral profile of the TMS click.^40^ A thin foam layer was placed between the coil and scalp to minimize bone-conducted auditory and somatosensory-evoked potentials. Participants were instructed to relax, minimize movement and fixate on a visual point on a screen.

### TMS–EEG preprocessing and analysis

EEG data were preprocessed using custom scripts in MATLAB (R2024a) with EEGLAB and the TMS–EEG Signal Analyzer (TESA) toolbox,^41^ following a previously described pipeline.^23^ The continuous EEG was epoched from −1400 ms to +1400 ms around each TMS pulse. We demeaned each epoch (subtracting its mean from all samples) instead of pre-stimulus baseline subtraction, as baseline correction before independent component analysis (ICA) can impair component estimation.^41^ Epochs with excessive noise or muscle artefacts were removed via visual inspection. The stimulation artefact was removed by cutting the signal from −5 to +10 ms relative to the TMS pulse, followed by interpolation of removed signal using a cubic spline, and downsampling to 1000 Hz. A first round of ICA (fastICA) was performed to remove muscle and TMS decay artefacts. The epochs were then filtered (1–100 Hz bandpass; 48–52 Hz notch, 4th order Butterworth) and trimmed to −1000 to +1000 ms to avoid edge effects. A second ICA round removed residual artefacts including ocular and muscle, and the data were re-referenced to the common average.

TEP components of interest were defined following a data-driven yet literature-guided approach. We analysed all canonical early TEPs up to 100 ms (P15, P30, N45, P60 and N100) to probe both local dynamics and network-level spread, while avoiding later components that are less well characterized and more susceptible to sensory co-stimulation confounds.^20,21^ First, a grand average across all participants was computed, and canonical M1 TEP components were identified with topographical plots within the following latency windows: 15–20 ms (P15), 25–35 ms (P30), 40–50 ms (N45), 55–65 ms (P60) and 90–110 ms (N100).^12^ The two electrodes showing the most positive (for P15, P30 and P60) or most negative (for N45 and N100) deflections within each window were selected as the regions of interest (ROIs). Selection of P15 over N15 was motivated by its association with transcallosal transmission.^42,43^ The average peak latencies were identified as the time point corresponding to the most positive (for P15, P30 and P60) or most negative (for N45 and N100) amplitude value of signal averaged within each ROI. The time windows of interest (TOIs) were then defined around the peaks as ±3 ms for P15, P30 and N45; ±5 ms for P60; and ±10 ms for N100. For each participant, TEP amplitudes were defined as the most positive (for P15, P30 and P60) or most negative (for N45 and N100) value within the ROI and TOI, and latency as the time point of this peak. Visual inspection of the grand-average waveform and topographies (see Supplementary material online, Fig. S1) confirmed the canonical M1 TEP profile. Specifically, P15 appeared as a centro-frontal positivity contralateral to stimulation, P30 as a central ipsilateral positivity, N45 as a contralateral negativity, P60 as a centro-parietal positivity and N100 as a centro-parietal negativity. The ROIs and TOIs for each component were average of Fz and FC2 at 15–21 ms (P15), of C3 and Cz at 28–34 ms (P30), of FCz and FC2 at 42–48 ms (N45), of C3 and CP5 at 50–60 ms (P60) and of C3 and CP1 at 93–113 ms (N100).

Time–frequency decomposition was performed from −1000 to +999 ms relative to the TMS pulse using complex Morlet wavelets with 45 logarithmically spaced frequencies (1–45 Hz), with variable number of cycles increasing from 3 to 10. Single-trial power was computed and normalized using a decibel (dB) baseline correction from −600 to −100 ms. Time–frequency representations were averaged across trials. A grand-average time–frequency matrix was computed across all participants and channels. Clusters of synchronization (relative power increase from baseline) and desynchronization (relative power decrease from baseline) were identified within the 15–400 ms post-stimulus as values above the 95th or below the 5th percentile of the decibel-normalized power distribution, respectively. From these clusters, rectangular windows were extracted, and we computed, for every channel, the average dB change, and ranked channels by absolute modulation. We then selected the smallest spatially contiguous set of channels whose mean modulation reached ≥90% of the local peak. This consistently yielded three-channel ROIs for all time–frequency windows. Based on this procedure, three TRSP variables were defined: Beta TMS-related synchronization (TRS) (17–21 Hz, 63–171 ms, channels C3, CP5, CP1), Gamma TRS (33–44 Hz, 15–55 ms, C3, CP1, FC1), and Gamma TMS-related desynchronization (TRD) (33–41 Hz, 210–399 ms, CP5, P7, P3) (see Supplementary material online, Fig. S2). For each subject, mean power was extracted from the relevant time–frequency window, averaged across the selected ROI electrodes.^44-46^ As an exploratory analysis, spatial focality was quantified within the three *a priori* TRSP windows defined above. For each participant and window we: (i) computed, for every electrode, the mean dB change within that window; (ii) formed a sign-aware peak mask including electrodes whose value reached ≥90% of that participant’s local extremum (maximum for TRS windows; minimum for the TRD window) and (iii) defined a focality index as: focality = (number of electrodes in the peak mask that fall within the grand-average ROI)/(total number of electrodes in the peak mask). Focality was set to 0 if the peak mask contained no ROI electrode. Higher values indicate peaks confined to the grand-average ROI, whereas lower values reflect more dispersed topographies.

### Statistical analysis

Statistical analyses were performed using SPSS (v.25) and RStudio (2024). Non-parametric comparisons between groups (HCs versus MS; NEDA versus EDA) were performed for each TEP component amplitude and latency, and on the three TRSP variables (Beta TRS, Gamma TRS and Gamma TRD) values and focality using Mann–Whitney U tests. Effect size (*r*) was computed as: |*Z*|/√*N* (*Z*, standardized test statistic; *N*, total observations). Correction for multiple comparisons was applied using the false discovery rate (FDR) method (controlled at *q* = 0.05). Spearman’s correlations were conducted between TMS and EEG measures that significantly differed between groups and clinical variables, including disease duration, EDSS and 9HPT scores of the right hand. In addition, a logistic regression analysis was performed to assess whether TMS–EEG measures predicted NEDA status at 2 years. First, univariate logistic regressions were conducted using the amplitude and latency of each TEP component. We planned to select those variables with a *P*-value of < 0.015 in univariate analysis to be included in a multivariate logistic regression model to identify independent predictors. A power analysis was conducted using G*Power (v.3.1.9.7) to determine the minimum sample size required for the primary comparison of the TEP amplitude between groups, using a non-parametric Mann–Whitney U test. In the absence of prior quantitative data on TEP amplitude comparison between MS and HCs, we selected an effect size of Cohen’s *d* = 0.7, reflecting a moderate-to-large effect, which we considered the minimum magnitude consistent with a functionally meaningful alteration in cortical excitability with potential clinical relevance. With power set at 80%, an *α* of 0.05 and assuming a group allocation ratio of 1.5:1 (MS:HC), chosen to support secondary within-group analyses in the MS cohort (e.g. NEDA versus EDA comparisons), the required total sample size was 58 participants (35 MS and 23 HCs).

## Results

### Demographic and clinical data

No significant differences in age (*t* = −0.84, *P* = 0.40) or sex distribution (*χ²* = 0.49, *P* = 0.49) were found between HCs (*n* = 26; 16 females/10 males, 35.7 ± 5.7 years) and MS patients (*n* = 43; 30 females/13 males, 37.5 ± 9.7 years). Within the MS group, disease duration at T0 had a median of 7 years (range 1–26), and the time since last clinical or radiological disease activity at T0 had a median of 24 months (range 1–177). Twelve MS patients were classified as EDA and 31 as NEDA at T1. Among the 12 patients classified as EDA, 8 experienced clinical relapses, 10 showed radiological activity (new or enlarging T2 lesions and/or gadolinium-enhancing lesions) and 6 exhibited EDSS worsening. EDA and NEDA subgroups did not differ in age (39.2 ± 7.2 versus 36.8 ± 10.6 years), sex (10 females/2 males versus 20 females/11 males) or disease duration [7 years (1–22) versus 7 years (1–26)]. However, EDSS at baseline (T0) was higher in the NEDA group (median 1.5) compared with EDA (median 1.0; U = 260, *P* = 0.04). Full details are reported in Table 1. RMT (MSO%) did not differ significantly between MS patients and HCs (69.15 ± 9.52 versus 69.77 ± 10.71; *t* = 0.24, *P* = 0.81) nor between the NEDA and EDA subgroups (69.71 ± 10.72 versus 69.92 ± 11.16; *t* = 0.06, *P* = 0.96). No MEPs were observed: across all subjects and analysed trials, right-FDI EMG time-locked to TMS remained <0.05 mV peak-to-peak post-TMS.

**Table 1:** Demographic and clinical characteristics of participants

Variable	HCs	MS total	HCs versus MS	MS EDA	MS NEDA	EDA versus NEDA
*N*	26	43	26 versus 43	12	31	12 versus 31
Age (mean ± SD)	35.7 ± 5.7	37.5 ± 9.7	*t* = −0.84, *P* = 0.40	39.2 ± 7.2	36.8 ± 10.6	*t* = −0.70 *P* = 0.491
Sex (F/M)	16/10	30/13	*χ²* = 0.49, *P* = 0.49	10/2	20/11	*χ²* = 1.45 *P* = 0.23
RMT, MSO% (mean ± SD)	69.15 ± 9.52	69.77 ± 10.71	*t* = 0.24, *P* = 0.81	69.71 ± 10.72	69.92 ± 11.16	*t* = 0.06 *P* = 0.96
Disease Duration, years [median (IQR), range]	—	7 (14), 1–26	—	7 (15), 1–22	7 (14), 1–26	U = 195 *P* = 0.80
EDSS T0 [median (IQR), range]	—	1.5 (2), 0–6.5	—	1.0 (2), 0–4.5	1.5 (1.5), 0–6.5	U = 260 ***P*** **=** **0.04**
EDSS T1 [median (IQR), range]	—	1.5 (2), 0–6.5	—	1.5 (1.5), 0–4.5	1.5 (2), 0–6.5	U = 197 *P* = 0.78
9HPT (mean ± SD)	—	22.5 ± 5.1	—	20.7 ± 3.6	23.3 ± 5.5	*t* = −1.45 *P* = 0.15
Clinical activity T1 (*n*)	—	8	—	8	—	—
Radiological act. T1 (*n*)	—	9	—	9	—	—
EDSS worsening T1 (*n*)	—	6	—	6	—	—
DMT (*n*)	—	CLA (9), DIM (2), NAT (30), OZA (1), TER (1)	—	CLA (3), DIM (2), NAT (5), OZA (1), TER (1)	CLA (6), NAT (25)	—

SD, standard deviation; F, female; M, male; HCs, healthy controls; MS, multiple sclerosis; EDA, evidence of disease activity; NEDA, no evidence of disease activity; RMT, resting motor threshold; MSO%, maximum stimulator output percentage; EDSS, Expanded Disability Status Scale; T0, baseline assessment; T1, 2-year follow-up assessment; 9HPT, 9-Hole Peg Test score for the right hand; DMT, disease-modifying therapy; CLA, cladribine; DIM, dimethyl fumarate; NAT, natalizumab; OZA, ozanimod; TER, teriflunomide. Statistical comparisons performed using independent samples *t*-tests (age, RMT and 9HPT), *χ*² test (sex) or Mann–Whitney U test (disease duration, EDSS). Data are presented as mean ± SD or median (range) as appropriate. Bold values denote statistical significance (*P* < 0.05).

### TEP results

**Figure 1:**
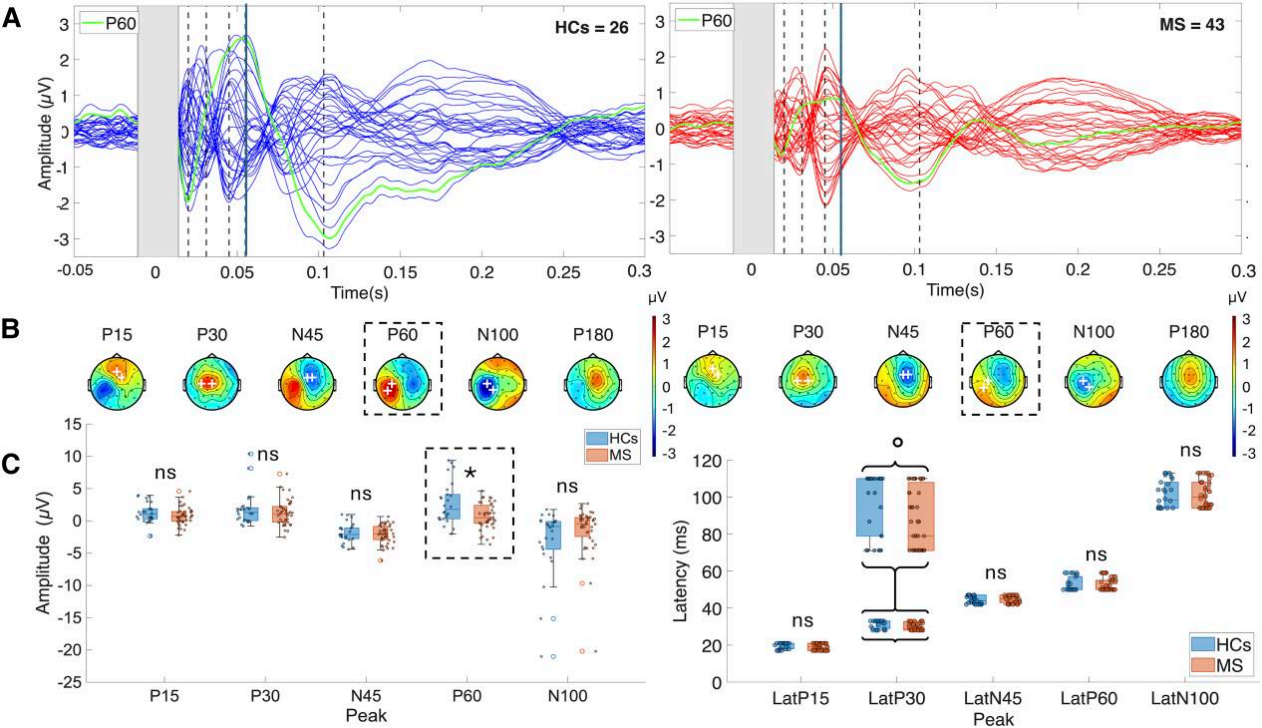
TEP comparison between MS patients and HCs. (**A**) Grand-average TEP waveforms elicited by left motor cortex stimulation in patients with MS (red) and HCs (blue). The grey rectangle represents interpolated EEG signal. Dashed vertical lines indicate the latency of the analysed TEP components, with the solid line marking the P60 peak, which showed a significant group difference. The green trace represents the mean TEP waveform within the ROI. (**B**) Scalp topographies of analysed TEP components for HCs (left) and MS (right). White crosses indicate the EEG channels included in the ROI. (**C**) Boxplots comparing TEP amplitude (left) and latency (right) between groups. Plots show median (horizontal line), interquartile range (box limits), range of non-outlier values (whiskers), outliers (circles) and single data values (dots). Statistical comparisons were performed using the Mann–Whitney U test. Significant differences were observed for P60 amplitude (U = 768, *P* = 0.0098, FDR-corrected *P* = 0.049) and P30 latency (U = 726.5, *P* = 0.031, FDR-corrected *P* = 0.153). Dashed outline indicates a statistically significant difference between groups. *P < 0.05 FDR-corrected; °*P* < 0.05 uncorrected; n.s., not significant; TEPs, TMS-evoked potentials.

**Figure 2:**
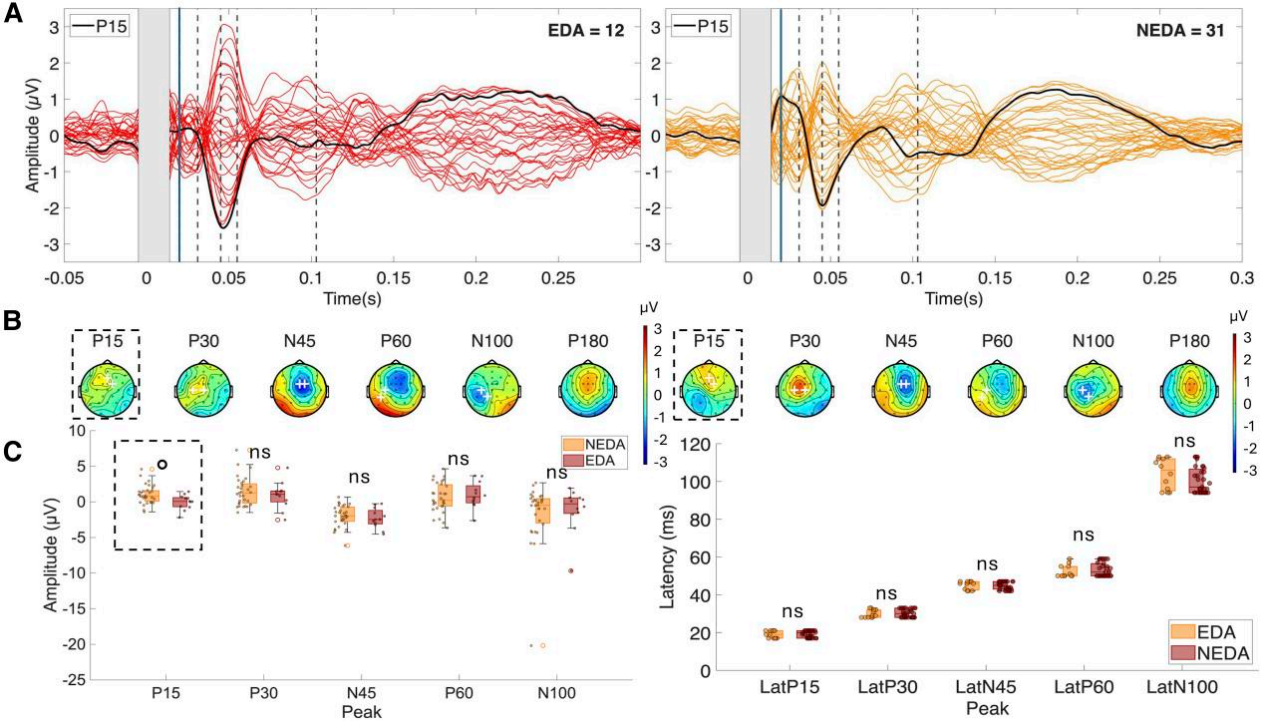
TEP comparison between EDA and NEDA patients with MS. (**A**) Grand-average TEP waveforms elicited by left motor cortex stimulation in patients with MS with EDA (red) and NEDA (orange). The grey rectangle represents interpolated EEG signal. Dashed vertical lines indicate the latency of the analysed TEP components, with the solid line marking the P15 peak, which showed a significant group difference. The black trace represents the mean TEP waveform within the ROI. (**B**) Scalp topographies of analysed TEP components for EDA (left) and NEDA (right). White crosses indicate the EEG channels included in the ROI. (**C**) Boxplots comparing TEP amplitude (left) and latency (right) between groups. Plots show median (horizontal line), interquartile range (box limits), range of non-outlier values (whiskers), outliers (circles) and single data values (dots). Statistical comparisons were performed using the Mann–Whitney U test. A trend-level difference was observed for P15 amplitude (U = 98, *P* = 0.0178, FDR-corrected *P* = 0.0891). Dashed outline indicates a statistically significant difference between groups. °*P* < 0.05 uncorrected; ns, not significant; TEPs, TMS-evoked potentials.

Visual inspection of butterfly plots (Fig. 1A) and scalp topographies (Fig. 1B) confirmed the presence of the canonical M1 TEP components in both groups. Peak presence was evaluated using the same ROIs and TOIs defined for amplitude/latency estimation (see the Materials and methods section). For each participant, we took the polarity-consistent extremum within the ROI/TOI and coded presence as 1 (clear local peak) or 0 for each TEP component. Peak presence (HCs versus MS) was P15, 81% versus 68%; P30, 92% versus 77%; N45, 89% versus 96%; P60, 69% versus 71%; and N100, 62% versus 58%. Two-sided Fisher’s exact tests showed no significant between-group differences (P15, *P* = 0.282; P30, *P* = 0.188; N45, *P* = 0.353; P60, *P* = 1.000; and N100, *P* = 0.623), indicating that the predefined narrow windows captured the intended peaks in the majority of participants in both groups. TEP components showed similar timing and distribution between MS patients and HCs, but the centro-parietal P60 positivity was visibly reduced in MS patients. Mann–Whitney U tests revealed a significant reduction in P60 amplitude in MS patients compared with HCs (U = 768, *P* = 0.0098, *r* = 0.312), which remained significant after FDR correction (*P* = 0.0491). No other components showed significant differences (Table 2 and Fig. 1C, left). NEDA participants showed higher P15 amplitude than EDA participants, though this effect was non-significant after FDR correction (U = 98, *P* = 0.0178, FDR-corrected *P* = 0.0891; *r* = 0.36). No other peaks showed significant or trend-level differences (Table 2 and Fig. 2). Mann–Whitney U tests showed a significant difference in the P30 latency that did not survive FDR correction (U = 727, *P* = 0.031, FDR-corrected *P* = 0.153, *r* = 0.091), with shorter latencies in MS patients. No other components showed significant latency differences between HCs and MS (Table 3 and Fig. 1C, right), nor between EDA and NEDA subgroups (Table 3 and Fig. 2C, right).

**Table 2:** Comparison of TMS-evoked potential amplitudes between groups

Peak	Median (IQR)	Median (IQR)	U	*P*	*P* adj. (FDR)	*r*
**HCs (26) versus MS (43)**	**HCs**	**MS**				
P15	1.07 (1.56)	0.71 (1.49)	669.0	0.1751	0.2919	0.164
P30	1.32 (1.91)	1.11 (2.38)	608.0	0.5481	0.6852	0.073
N45	−2.12 (1.63)	−2.00 (2.01)	568.0	0.9161	0.9162	0.013
P60	1.79 (3.69)	0.49 (2.73)	768.0	**0**.**0098**	**0**.**0491**	0.312
N100	−0.88 (4.20)	−0.48 (2.72)	427.0	0.1034	0.2586	0.197
**EDA (12) versus NEDA (31)**	**EDA**	**NEDA**				
P15	0.07 (1.21)	0.81 (1.36)	98.0	**0**.**0178**	0.0891	0.363
P30	1.08 (1.40)	1.25 (2.64)	164.0	0.5605	0.7006	0.091
N45	−2.47 (1.65)	−1.95 (1.92)	156.0	0.4244	0.7006	0.124
P60	0.72 (2.00)	0.22 (2.95)	212.0	0.4899	0.7006	0.107
N100	−0.27 (2.12)	−0.48 (3.29)	197.0	0.7762	0.7762	0.045

TEP, TMS-evoked potential; HCs, healthy controls; MS, multiple sclerosis; EDA, evidence of disease activity; NEDA, no evidence of disease activity; IQR, interquartile range; U, Mann–Whitney U statistic; *P* adj. (FDR), *P*-value adjusted for multiple comparisons using the FDR method; *r*, effect size computed as *r* = |*Z*|/√*N*. Data represent median (IQR) TEP amplitude in microvolts. Statistical comparisons performed using Mann–Whitney U test. Bold values denote statistical significance (*P* < 0.05).

**Table 3:** Comparison of TMS-evoked potential latencies between groups

Peak	Median (IQR)	Median (IQR)	U	*P*	*P* adj. (FDR)	*r*
**HCs (26) versus MS (43)**	**HCs**	**MS**				
P15	21 (18–21)	19 (17–21)	642.0	0.270	0.675	0.134
P30	33 (29–33)	29 (28–32)	726.5	**0**.**031**	0.153	0.261
N45	44 (42–47)	45 (43–47)	497.5	0.435	0.726	0.095
P60	50 (50–56)	50 (50–55)	535.0	0.751	0.795	0.038
N100	98 (94–108)	100 (94–108)	538.0	0.795	0.795	0.032
**EDA (12) versus NEDA (31)**	**EDA**	**NEDA**				
P15	19 (17–21)	20 (17–21)	169.5	0.644	0.713	0.073
P30	29 (28–32)	30 (28–33)	172.5	0.713	0.713	0.058
N45	46 (43–47)	45 (43–47)	200.5	0.693	0.713	0.062
P60	50 (50–55)	52 (50–56)	163.5	0.521	0.713	0.100
N100	106 (96–112)	97 (94–106)	234.0	0.190	0.713	0.202

TEP, TMS-evoked potential; HCs, healthy controls; MS, multiple sclerosis; EDA, evidence of disease activity; NEDA, no evidence of disease activity; IQR, interquartile range; U, Mann–Whitney U statistic; *P* adj. (FDR), *P*-value adjusted for multiple comparisons using the FDR method; *r*, effect size computed as *r* = |*Z*|/√*N*. Data represent median (IQR) TEP latency in millisecond. Statistical comparisons performed using Mann–Whitney U test. Bold values denote statistical significance (*P* < 0.05).

### TRSP results

**Figure 3:**
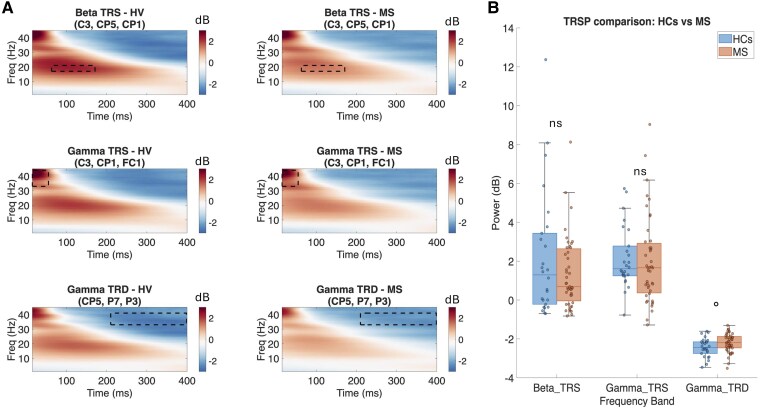
TRSP comparison between MS patients and HCs. (**A**) Group-level time–frequency representations obtained by averaging subject-level data (HCs, *N* = 26; MS, *N* = 43). Beta TRS (*top*), Gamma TRS (*middle*) and Gamma TRD (*bottom*) in HCs (left) and MS patients (right), averaged over the respective ROI electrodes. Power is expressed in decibels (dB), baseline-corrected using the −600 to −100 ms pre-stimulus interval. Dashed rectangles indicate the time–frequency windows used for group comparisons. (**B**) Boxplots comparing mean power (dB) for each TRSP variable between groups. Plots show median (horizontal line), interquartile range (box limits), range of values (whiskers) and single data values (dots). Statistical comparisons were performed using the Mann–Whitney U test. Gamma TRD showed a significant uncorrected difference (U = 740, *P* = 0.025, FDR-corrected *P* = 0.075). No significant differences were found for Beta TRS (U = 522, *P* = 0.647) or Gamma TRS (U = 489, *P* = 0.386). °*P* < 0.05 uncorrected; n.s., not significant. TRSP, TMS-related spectral perturbations; TRS, TMS-related spectral synchronization; TRD, TMS-related spectral desynchronization.

**Figure 4:**
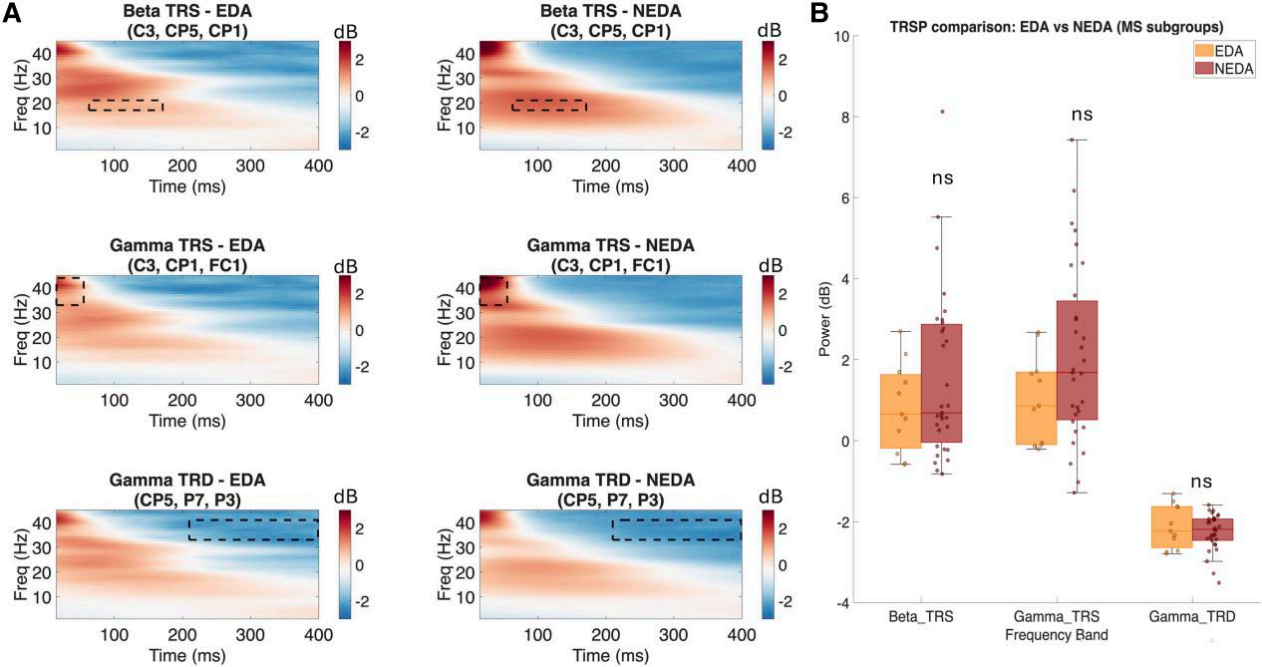
TRSP comparison between EDA and NEDA patients with MS. (**A**) Group-level time–frequency representations obtained by averaging subject-level data (EDA, *N* = 12; NEDA, *N* = 31). Beta TRS (*top*), Gamma TRS (*middle*) and Gamma TRD (*bottom*) in patients with MS with EDA (left) and NEDA (right), averaged over the respective ROI electrodes. Power is expressed in decibels (dB), baseline-corrected using the −600 to −100 ms pre-stimulus interval. Dashed rectangles indicate the time–frequency windows used for group comparisons. (**B**) Boxplots comparing mean power (dB) for each TRSP variable between groups. Plots show median (horizontal line), interquartile range (box limits), range of values (whiskers) and single data values (dots). Statistical comparisons were performed using the Mann–Whitney U test. No significant differences were observed for Beta TRS (U = 213, *P* = 0.478), Gamma TRS (U = 218, *P* = 0.399) or Gamma TRD (U = 163, *P* = 0.547). n.s., not significant; TRSP, TMS-related spectral perturbations; TRS, TMS-related spectral synchronization; TRD, TMS-related spectral desynchronization.

Visual inspection of time–frequency plots (Figs 3A and 4A) revealed a consistent pattern of TMS-induced spectral perturbations across groups, characterized by predominant early beta- and gamma-band synchronization followed by desynchronization over the stimulated motor cortex, and less prominent alpha- and theta-band synchronization. Scalp topographies (see Supplementary material online, Fig. S3) showed that the spatial distribution of TRSPs was overall similar between groups. Specifically, Beta TRS was centred over left centro-parietal regions, Gamma TRS over fronto-central areas and Gamma TRD over left posterior parietal regions (see Supplementary material online, Fig. S3). Exploratory analysis on TRSP focality showed no significant differences between MS and HC for Beta TRS (*P* = 0.209) or Gamma TRS (*P* = 0.862), with a trend towards greater focality in HCs for Gamma TRD (*P* = 0.055). Within MS, EDA and NEDA did not differ for Beta TRS (*P* = 0.880), Gamma TRS (*P* = 0.413) or Gamma TRD (*P* = 0.899) focality. Mann–Whitney U tests comparing MS and HC revealed a reduction in Gamma TRD (i.e. less negative values) in MS patients compared with HCs, though this difference was non-significant after FDR correction (U = 740, *P* = 0.025, *P* adj. = 0.075, *r* = 0.270). Beta TRS (U = 522, *P* = 0.647 *P* adj. = 0.647, *r* = 0.055) and Gamma TRS (U = 489, *P* = 0.386 *P* adj. = 0.579, *r* = 0.104) showed no significant differences between groups (see Supplementary material online, Fig. S3). No significant differences were observed in Beta TRS (U = 213, *P* = 0.478, *P* adj. = 0.547, *r* = 0.111), Gamma TRS (U = 218, *P* = 0.399, *P* adj. = 0.547, *r* = 0.132) nor Gamma TRD (U = 163, *P* = 0.547, *P* adj. = 0.547, *r* = 0.095) between EDA and NEDA subgroups (see Supplementary material online, Fig. S4).

### Correlation analyses

**Figure 5:**
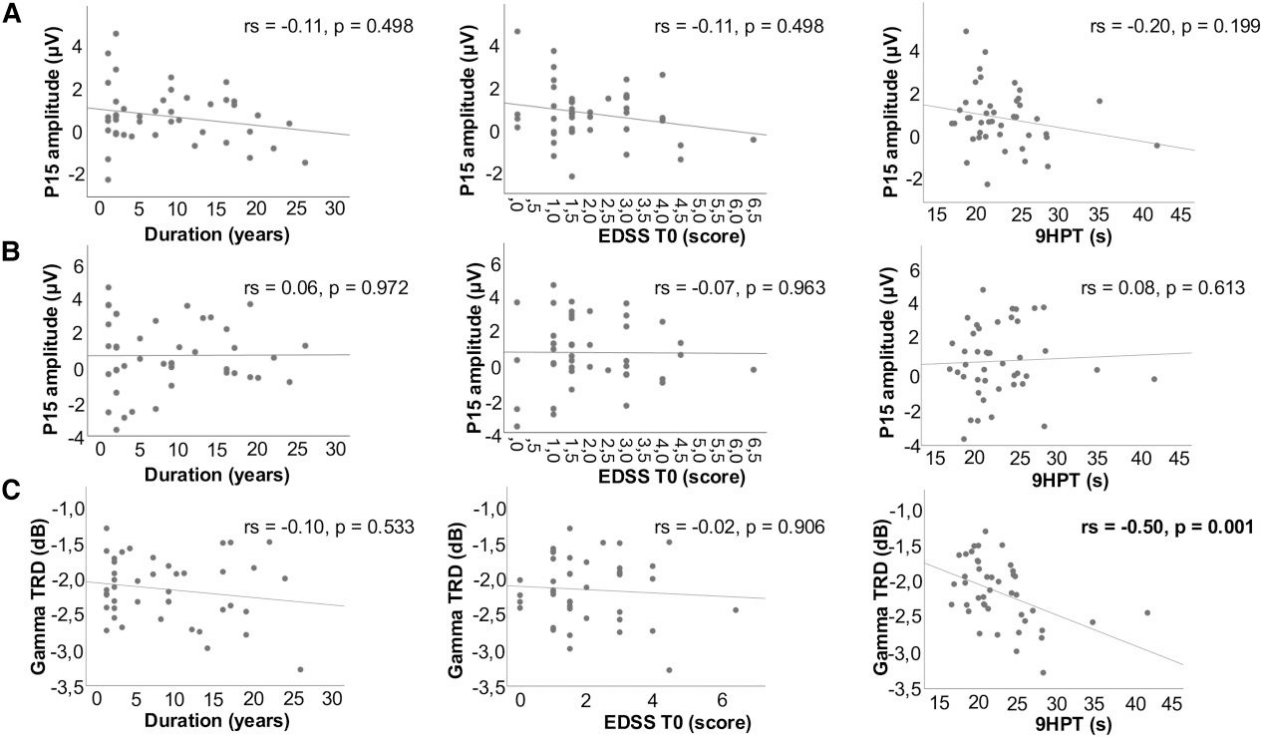
Correlation between TEPs and TRSP and clinical variables in MS patients. (**A**) Scatterplots showing the distribution of P15 amplitude values as a function of disease duration (left), EDSS at baseline (centre) and 9HPT performance (right). (**B**) Scatterplots showing the distribution of P60 amplitude values as a function of the same clinical variables. (**C**) Scatterplots showing the distribution of gamma-band TRD values as a function of the same clinical variables. Each dot represents an individual patient. The grey line indicates a linear fit provided for visualization purposes only. Spearman’s *ρ* (*r*_s_) and corresponding *P*-values are reported in each panel. TEP, TMS-evoked potential; TRSP, TMS-related spectral perturbations; TRD, TMS-related desynchronization; EDSS, Expanded Disability Status Scale; 9HPT, 9-Hole Peg Test; MS, multiple sclerosis.

No significant correlations were found between the TEP components that differentiated between groups and clinical variables (Fig. 5). Specifically, P60 amplitude (which differed between HCs and MS patients) and the P15 amplitude (examined despite a non-significant NEDA versus EDA difference but with a moderate effect size) showed no significant correlation with disease duration, EDSS at T0 or right-hand 9HPT performance (all *P* > 0.19). The gamma-band TRD correlated negatively with 9HPT times (*r*_s_ = −0.504, *P* = 0.001), indicating that less-pronounced desynchronization (i.e. less negative TRD values) was associated with faster 9HPT performance; no associations were observed with disease duration or baseline EDSS (all *P* > 0.5).

Prompted by a reviewer, we explored in MS patients the relationship between TEPs and TRSP in those features different from HCs (see Supplementary material online, Fig. S5). Spearman’s rank correlation analysis showed significant positive associations between the TEP P15 amplitude and Beta TRS (*r*_s_ = 0.32, *P* = 0.036), Gamma TRS (*r*_s_ = 0.40, *P* = 0.008), but not with Gamma TRD (*r*_s_ = 0.19, *P* = 0.213), and between TEP P60 amplitude and Beta TRS (*r*_s_ = 0.45, *P* = 0.003), but not with Gamma TRS (*r*_s_ = 0.14, *P* = 0.371) or Gamma TRD (*r*_s_ = −0.20, *P* = 0.205).

### Regression analysis

Among the univariate logistic regression models evaluating the predictive value of TEP amplitude and latency, and TRSP for NEDA status at 2-year follow-up, only P15 amplitude met the inclusion threshold (*P* < 0.10); therefore, a multivariate model was not performed (see Supplementary material online, Table S1). A higher P15 amplitude was associated with increased odds of achieving NEDA-3 [Exp(*B*) = 2.321, 95% CI (1.121–4.807), *P* = 0.023] (see Supplementary material online, Fig. S4A). The univariate model based on P15 alone yielded an overall classification accuracy of 74.4%, with a sensitivity of 93.5% and a specificity of 25.0% (see Supplementary material online, Fig. S4B). Given the class imbalance (NEDA = 31 and EDA = 12), we also computed a balanced accuracy (sensitivity + specificity/2) of 59.5%. The ROC analysis revealed an area under the curve (AUC) of 0.737 (95% CI 0.581–0.892; *P* = 0.017). The optimal cut-off for P15 amplitude was identified at 0.53 µV (Youden index), yielding a sensitivity of 71% and a specificity of 75%.

## Discussion

This study provides new evidence that TMS–EEG captures functional alterations in specific TMS–EEG measures of motor cortical excitability in people with relapsing–remitting MS. Specifically, we found a significant reduction in the P60 component amplitude and a trend for a reduction in gamma-band desynchronization following M1 stimulation in MS patients compared with HCs. Furthermore, we observed a trend towards reduced P15 amplitude in MS patients with disease activity (EDA) versus without disease activity (NEDA) at 2-year follow-up. Importantly, the P15 amplitude was associated with longitudinal disease stability as measured by NEDA-3 status; however, this result arose under marked class imbalance (31 NEDA versus 12 EDA) and showed low specificity, so it should be interpreted cautiously.

Our results underscore the power of TMS–EEG to directly probe motor cortical excitability, extending MEP-based evidence of corticospinal dysfunction in MS.^9-11^ TMS–EEG isolates cortical responses, offering a more specific view of intrinsic motor network dynamics unaffected by downstream corticospinal transmission confounders related to MEPs.^12^ In our study, subthreshold stimulation of the left M1 elicited in both MS and HCs a sequence of TEPs with a spatial and temporal profile consistent with canonical M1 responses previously described in the literature.^47,48^ This observation reinforces the notion that, when appropriate methodological precautions are adopted—namely subthreshold stimulation at 90% RMT, time-locked auditory masking with continuous background noise, coil cushioning and a standardized TESA-based dual-ICA preprocessing (see the Materials and methods section)—TMS–EEG yields lateralized, area-specific responses over the stimulated sensorimotor areas. Such characteristics support the use of TMS-evoked responses as a genuine readout of the functional state of the targeted cortex rather than artefactual co-stimulation, in line with prior evidence.^20^ Although mean amplitudes showed the expected polarity for each component, TEP waveforms exhibited substantial inter-subject variability, which is in line with previous studies.^49^ Despite this variability, our group-level inference is robust because we average within ROIs and use rank-based (Mann–Whitney) tests that are relatively insensitive to outliers and sign inversions. At present, only one study has investigated TEP abnormalities at rest in MS patients compared with HCs^22^ and found no significant differences in early TEP components between groups. Several factors may account for the discrepancies of this previous study with our study, including the exclusive inclusion of early, low-disability patients, the exclusion of early components (N15/P30) due to TMS artefact contamination and the use of a stimulation intensity of 100% RMT potentially inducing corticospinal activation and sensory reafference.^50^ Additionally, MS patients in Zipser *et al*.^22^ study had higher RMTs than controls, resulting in higher absolute stimulation intensities that may have masked subtle group differences, particularly in components that do not scale linearly with intensity.

While we observed significant group differences in the amplitude of TEP P60 component, no differences in peak latency were detected. This finding suggests that the observed alterations in TEPs are unlikely to be driven by slowed conduction and chronodispersion due to demyelination.^51^ Instead, amplitude changes in TEPs may reflect functional disturbances in synaptic efficacy, excitation/inhibition balance or cortico-cortical and thalamo-cortical network dynamics.^4-7,52^ Therefore, the observed reduction in the P60 amplitude in MS patients may reflect impaired motor cortical excitability or disrupted motor network dynamics. Although its origin is not yet fully understood, the P60 component evoked by M1 stimulation is predominantly generated in the ipsilateral somatosensory cortex and thus likely reflects recurrent cortico-cortical or cortico-subcortico-cortical processing within the sensorimotor network.^53^ While previous studies have shown that the P60 amplitude can be modulated by somatosensory feedback arising from peripheral muscle activation with TMS intensities at or above motor threshold,^50^ we stimulated below motor threshold, precluding this confound. At the subthreshold intensity used here, our stimulation likely predominantly recruited trans-synaptic intracortical networks (I-waverelated circuits) and reverberant cortico-cortical/thalamo-cortical loops.^54^ Pharmacological work shows that α-amino-3-hydroxy-5-methyl-4-isoxazoleproprionic acid (AMPA) receptor blockade resulted in depression of the P60 amplitude,^15^ but AMPA is unlikely to be the sole determinant. At subthreshold intensities, P60 amplitude could also be shaped by the excitatory–inhibitory balance in local microcircuits (e.g. fast GABA_A-mediated inhibition constraining recurrent activity) and by thalamo-cortical drive that gates sensory–motor integration. Consistent modulation of P60 by interventions targeting intracortical excitability^14,55-57^ and sensorimotor plasticity^58,59^ further supports this network interpretation. Therefore, P60 attenuation in MS may reflect disrupted sensorimotor excitability and connectivity, potentially reflecting impaired cortico-cortical and subcortico-cortical dynamics.

From a clinical standpoint, a notable preliminary observation in our study was the association between higher P15 amplitude and achievement of NEDA-3 status at 2 years. The P15 component has been linked in healthy individuals to early interhemispheric signal transmission.^42,43^ In particular, Bortoletto *et al*.^42^ showed that the P15 latency is predicted by callosal microstructural properties, while P15 amplitude correlates with the strength of transcallosal inhibition. In our study, the absence of group differences in P15 latency suggests preserved structural timing of interhemispheric transmission, whereas the association between reduced P15 amplitude and failure to achieve NEDA-3 points to a selective functional impairment in the efficacy of transcallosal inhibition, rather than a delay in conduction *per se*. Thus, reduced P15 amplitude may represent an early, functionally relevant marker of network disinhibition or reduced interhemispheric integration capacity in MS rather than demyelination of the corpus callosum. We do not claim a causal relation between baseline P15 and future disease activity. In this study, the P15–NEDA association should be regarded as a proxy analysis: NEDA-3 is a coarse, composite indicator of overall stability (relapses, MRI activity, EDSS) rather than a network-specific end-point; we used it as a pragmatic clinical summary to explore whether an electrophysiological marker of preserved interhemispheric function aligns with a globally ‘stable’ course. This remains hypothesis-generating and requires confirmation in balanced cohorts with prespecified thresholds. The association between higher P15 amplitude and disease stability suggests that this measure could serve as a marker of preserved motor network function; however, any risk-stratification use is premature and will require validation in balanced cohorts with prospective thresholds and preregistered analyses.

Although our findings suggest that TEPs from M1 distinguished MS patients from HCs, we found no significant correlations with disease duration, EDSS or 9HPT performance, despite the presence of marked motor impairments in several patients. This dissociation may reflect the fact that TEP P60 reduction captures subclinical dysfunction or functional abnormalities that do not directly translate into overt motor impairment. This apparent lack of correlation may simply reflect a construct mismatch: the P60 component indexes the physiology of a spatiotemporally precise motor–cortical circuit, whereas EDSS and 9HPT are composite behavioural indices that aggregate performance across multiple systems.^60^ In this framework, M1 TEP alterations may represent functional proxies of motor network-level dysfunction, capturing subtle physiological changes that do not yet translate into overt disability (i.e. not detectable through standard clinical tools). Alternatively, as we only sampled a limited set of functions, P60 abnormality could relate to motor disturbances not captured by the chosen readouts (e.g. fatigability, effort–cost and fine force control). Concomitant TMS–EEG and MRI studies are needed to clarify how TMS–EEG alterations relate to established structural and functional MRI markers and to determine where these functional disturbances fit within the broader pathophysiological landscape of MS. It will also be important to assess whether TEP alterations can predict disability progression and motor performance decline in longitudinal studies, thereby supporting their utility as prognostic biomarkers.^61^

Beyond TEPs, TMS–EEG also enables the investigation of TRSP, which provides complementary insights into the functional architecture of the stimulated oscillatory networks.^26-28,30^ In the current study, TMS applied to M1 resulted in early synchronization and late desynchronization of EEG oscillations, particularly in the alpha, beta and gamma bands, near the stimulation site, consistent with TRSP previously described in the literature.^30,62-64^ In MS patients, while the overall spatiotemporal profile of TRSPs was preserved, we observed a trend towards a selective reduction in gamma-band desynchronization (i.e. less negative values) following M1 stimulation, both in terms of magnitude and in focality, suggesting a possible alteration in high-frequency oscillatory dynamics. Gamma-band oscillations are thought to reflect local cortical processing, fine-scale interneuronal synchronization, and dynamic gating of motor output, largely mediated by fast-spiking GABAergic interneurons.^65^ Whereas the immediate synchronization following TMS likely reflects transient activation and/or phase reset of oscillatory cortico-cortical and cortico-subcortical circuits, the subsequent desynchronization may reflect thalamo-cortical network reorganization or disengagement from the perturbed state.^26,29,66^ Albeit the significance of the late desynchronization remains unclear, pharmacological TMS–EEG evidence suggests that it may arise from GABAergic inhibitory mechanisms.^63^ Interesting though, within the MS cohort we found that reduced gamma-band desynchronization (i.e. less negative values) correlated with and finer motor dexterity (i.e. lower 9HPT values). This pattern suggests that an attenuated gamma desynchronization may represent a plastic, homeostatic ‘fine-tuning’ of inhibition rather than a mere loss of function. By reducing post-stimulus suppression, the cortical networks could regain operational efficiency and preserve performance despite structural damage, making reduced gamma desynchronization a potential marker of functional reserve. Altogether, these findings suggest that gamma-band TRSP alterations may provide physiologically meaningful biomarkers of motor network adaptive plasticity in MS, with potential relevance for guiding individualized neuromodulatory interventions.^34,35^ Exploratory analyses showed that in MS patients, the early P15 peak correlates with fast, gamma-band synchronization, while the intermediate P60 peak correlates with beta-band synchronization. Notably, these within-subject associations did not translate into TRSP differences between groups, underscoring that TEP–TRSP coupling is detectable at the individual level but does not necessarily drive group-level variation. One possible explanation is that phase-locked TEPs capture specific evoked responses, while TRSP reflects a mixture of phase-locked and non-phase-locked oscillatory changes.^27^ Overall, these findings point to phase-locked activity, embodied by P15 and P60, as a potentially more sensitive indicator of MS-related sensorimotor dysfunction. The precise mechanistic significance of these response components, however, remains speculative and warrants dedicated, hypothesis-driven investigation.

Several limitations must be acknowledged. First, although the study was adequately powered for the prespecified primary MS versus HC comparison, the EDA versus NEDA subgroup contrasts were relatively small and may be underpowered, potentially obscuring smaller effects and limiting the generalizability of those specific findings. Although the P15 component occurs close to the offset of the TMS artefact and may be susceptible to contamination by muscular artefacts, several arguments support its cortical origin. Muscle artefacts typically manifest as high-amplitude biphasic deflections with a peripheral scalp distribution ipsilateral to stimulation, whereas the P15 observed here displays a tangential centro-frontal dipolar topography contralateral to the stimulated M1, which is not consistent with peripheral muscle activity (Figs 1B and 2B). This cortical attribution was likely made feasible by our methodological choices—a DC-coupled, high-bandwidth system sampled at 10 kHz to keep the TMS artefact brief, subthreshold motor stimulation to limit muscle activity and two ICA passes to effectively suppress residual artefact. Moreover, because acquisition settings and artefact-removal procedures were identical across groups, and RMT did not differ between NEDA and EDA patients (making muscle twitches equally likely in both), the possibility that residual muscle artefacts systematically biased the two subgroups can be discounted. Another technical consideration is the complexity of TMS–EEG data processing; despite following established best practices,^41^ some residual effects of the TMS pulse or muscle activity might linger and/or the physiological signal could have been over-corrected. As more automated and standardized pipelines are developed, the reliability of early component detection (like P15) will further improve. Given the high sensitivity but low specificity (balanced accuracy ≈0.59) of our NEDA prediction model, a larger P15 amplitude may help flag patients likely to remain stable, whereas a smaller amplitude does not reliably indicate progression risk; accordingly, this finding should be treated as hypothesis-generating and requires confirmation in larger, balanced, preregistered cohorts. The absence of structural imaging correlates (e.g. lesion load or cortical atrophy) limits our ability to link functional findings to anatomical substrates. In this study, disease activity was operationalized dichotomously based on the NEDA-3 criteria, which, although widely used, may not fully capture the complexity of disease progression and is not tailored to network-specific dysfunction. Future studies should adopt a more granular characterization of clinical status, including patient-reported outcomes, explicitly account for treatment exposure and lesion topology, and test associations with fluid inflammatory biomarkers. This approach may enhance the interpretability of TMS–EEG alterations and clarify their functional significance within the broader spectrum of disease burden. Despite employing state-of-the-art sensory suppression techniques in our TMS–EEG measurements, later TEP components may be partially contaminated by sensory co-stimulation.^25^ However, supporting the validity of our approach, the late TEP components in our data were of relatively low amplitude and showed a clear lateralized distribution, consistent with a predominantly cortical origin. Moreover, if the observed group difference in the P60 amplitude merely reflected altered processing of the sensory co-stimulation in MS patients, one would expect even greater differences in later, more sensory-dominated components such as the N100, yet this was not the case. This further supports the interpretation of the P60 amplitude as a marker of altered cortical excitability or network integrity rather than a spurious effect of contamination of this TEP component by sensory input. Moreover, sham TMS experiments have provided strong evidence that the early TEP components with latencies <70 ms after the TMS pulse are not significantly contaminated by sensory input but rather reflect specific brain responses to TMS.^20,48,67^ Finally, baseline EDSS was higher in NEDA despite similar disease duration across subgroups; this likely reflects earlier disability accumulation followed by escalation to high-efficacy therapy (e.g. natalizumab) and subsequent stability, rather than a direct link between NEDA status and greater disability. Accordingly, baseline EDSS and therapy class should be considered potential confounders, and larger balanced cohorts are needed to control for these factors.

This study demonstrates the feasibility and potential value of TMS–EEG for detecting MS-related alterations in specific network-level measures of motor cortical excitability. Overall, our findings point to a robust MS-related reduction of P60 as the primary group effect and of gamma-band desynchronization exhibiting an uncorrected trend, with P15 showing an exploratory association with longitudinal stability (NEDA-3), together delineating targeted rather than global changes in motor network excitability. By providing direct, temporally precise measures of cortical reactivity and effective connectivity, TMS–EEG complements resting-state fMRI evidence of sensorimotor network dysfunction and offers unique insights into the dynamic mechanisms of network disruption not accessible through haemodynamic-based imaging.^68^ These features position TMS–EEG as a highly promising bridge between structural pathology and clinical expression of disease. Future studies should aim to replicate these findings in larger, independent cohorts, assess the test–retest reliability and generalizability of TMS–EEG measures and explore their sensitivity to treatment effects. Longitudinal and multimodal approaches combining TMS–EEG with advanced MRI metrics are warranted to clarify the structural–functional correlates of TEP and TRSP alterations and to refine their prognostic value. In conclusion, TMS–EEG emerges as a powerful non-invasive tool to probe cortical function in MS.

## Supplementary Material

fcag028_Supplementary_Data

## Data Availability

The data that support the findings of this study are available on request from the corresponding author.
