# Supplementary material for: TMS–EEG signatures of motor network dysfunction in multiple sclerosis
Source: Brain Commun. 2026 Jan 31;8(1):fcag028. doi: 10.1093/braincomms/fcag028 (PMC12914579; doi:10.1093/braincomms/fcag028)
Supplement: fcag028_Supplementary_Data [file fcag028_supplementary_data.pdf]

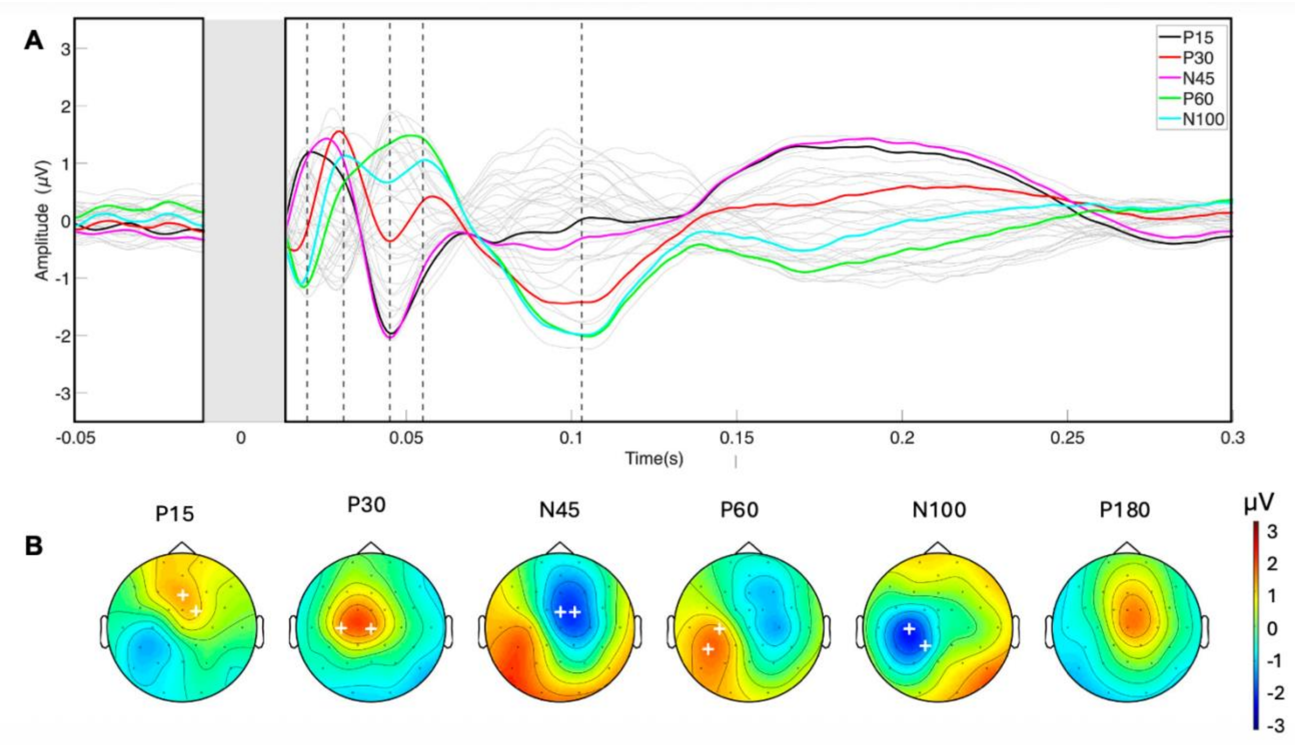

Supplementary Figure 1. Grand-average TEPs and identification of components

(**A**) Grand-average (all subjects) butterfly plot across all participants showing TEP waveforms across all EEG channels (gray lines). Colored lines represent the average waveform computed across the two electrodes selected as the region of interest (ROI) for each canonical TEP component: P15 (black), P30 (red), N45 (magenta), P60 (green), and N100 (cyan). Dashed vertical lines indicate the latency windows used for component identification. The shaded gray area corresponds to the TMS artifact window, excluded from analysis.

(**B**) Scalp topographies of each TEP component, averaged within the respective time windows of interest (TOIs): P15 (15–21 ms), P30 (28–34 ms), N45 (42–48 ms), P60 (50–60 ms), and N100 (93–113 ms). TEP = TMS-evoked potential; M1 = primary motor cortex.

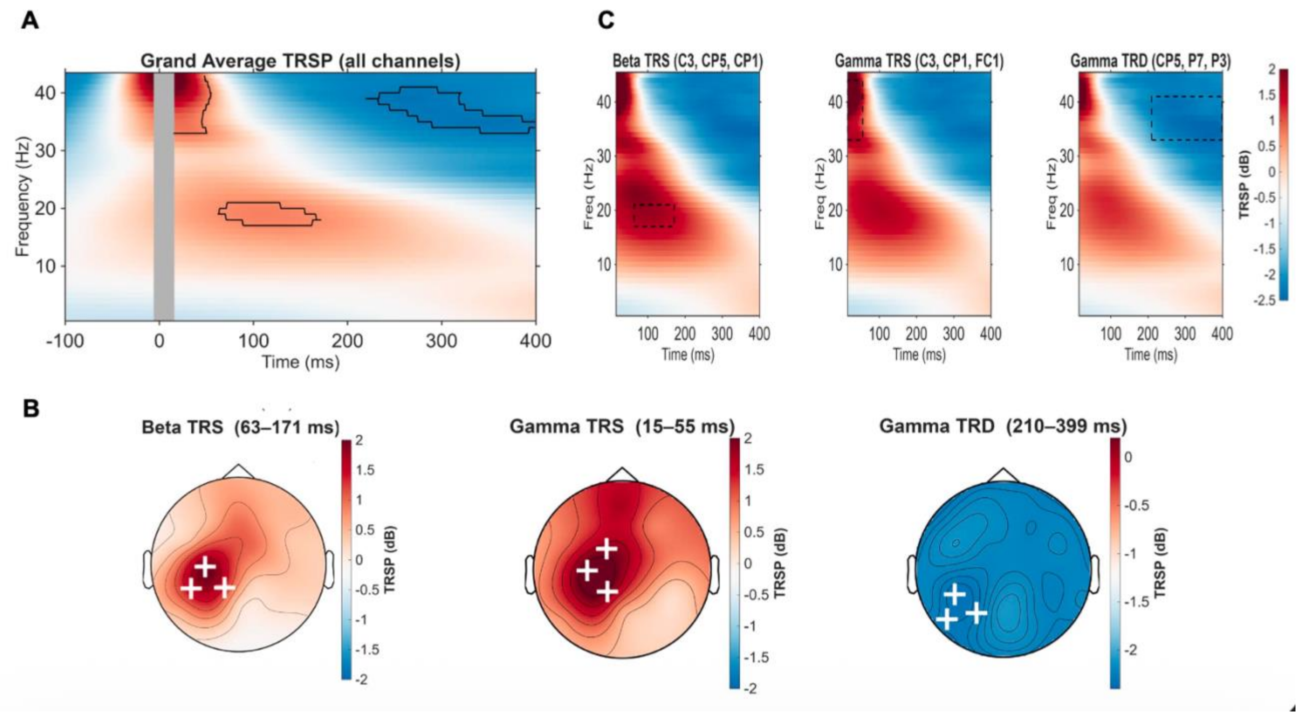

Supplementary Figure 2. **Grand-average TRSP and identification of time-frequency clusters**

(**A**) Grand-average time–frequency representation of TRSP across all channels and participants, baseline-corrected in decibels (dB) relative to the –600 to –100 ms pre-stimulus interval. Black contours mark clusters of significant synchronization and desynchronization identified within the 15–400 ms post-TMS window, used to define TRSP features.

(**B**) Scalp topographies of each TRSP variable, averaged over their respective time windows of interest (TOIs). Crosses indicate the three electrodes showing maximal (for TRS) or minimal (for TRD) power modulation, selected as regions of interest (ROIs).

(**C**) Time–frequency maps from the selected ROI channels (indicated in parentheses), showing the extracted time-frequency clusters (dashed rectangles) used to extract each TRSP variable for subject-level quantification. TRSP = TMS-related spectral perturbation; TRS = TMS-related synchronization; TRD = TMS-related desynchronization.

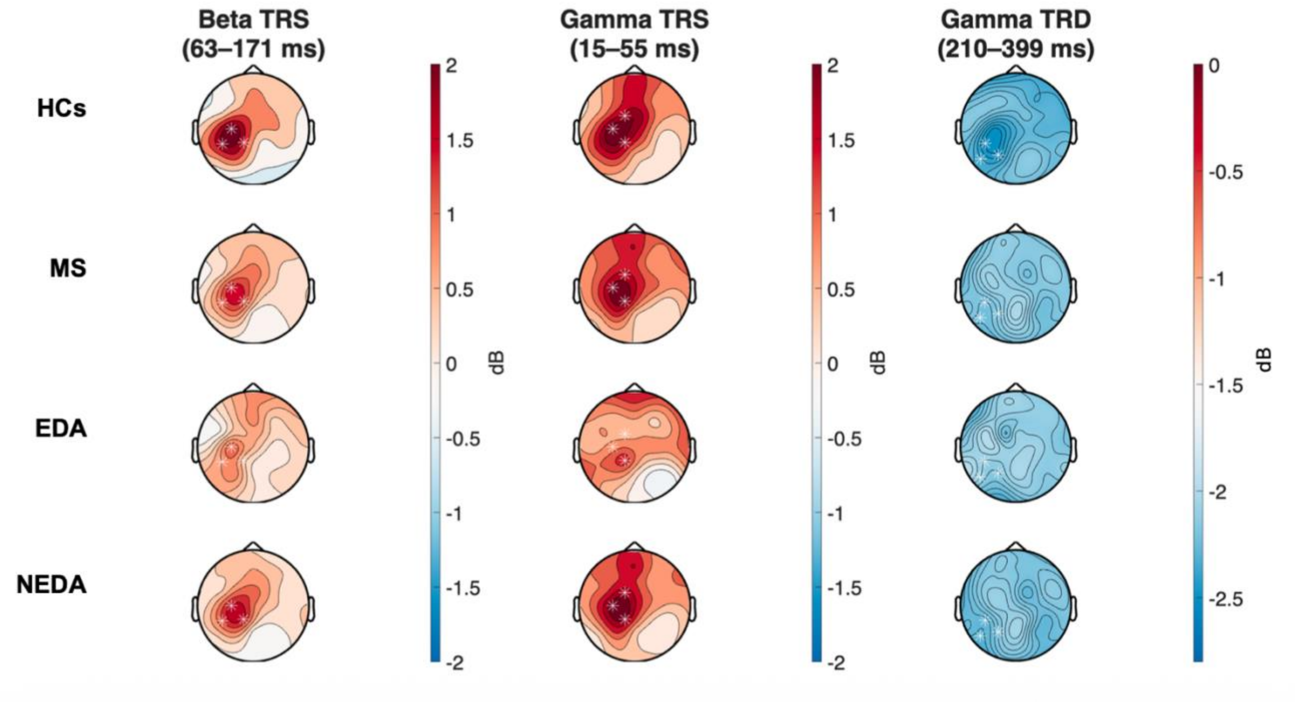

Supplementary Figure 3. Topographical distribution of TRSPs across groups

Topographical maps show beta and gamma-band TMS-related spectral perturbations (TRSPs) in healthy controls (HCs), all MS patients (MS), and subgroups with (EDA) or without (NEDA) evidence of disease activity. Each column corresponds to a TRSP variable derived from data-driven time–frequency clusters: beta-band synchronization (TRS), early gamma-band synchronization (Gamma TRS), and late gamma-band desynchronization (Gamma TRD). The time ranges in parentheses indicate the post-TMS intervals over which power was averaged. Asterisks mark the three electrodes within each time-frequency cluster showing the strongest TRSP, i.e. maximal power for synchronization, minimal for desynchronization, used as regions of interest (ROIs) for group-level comparisons.

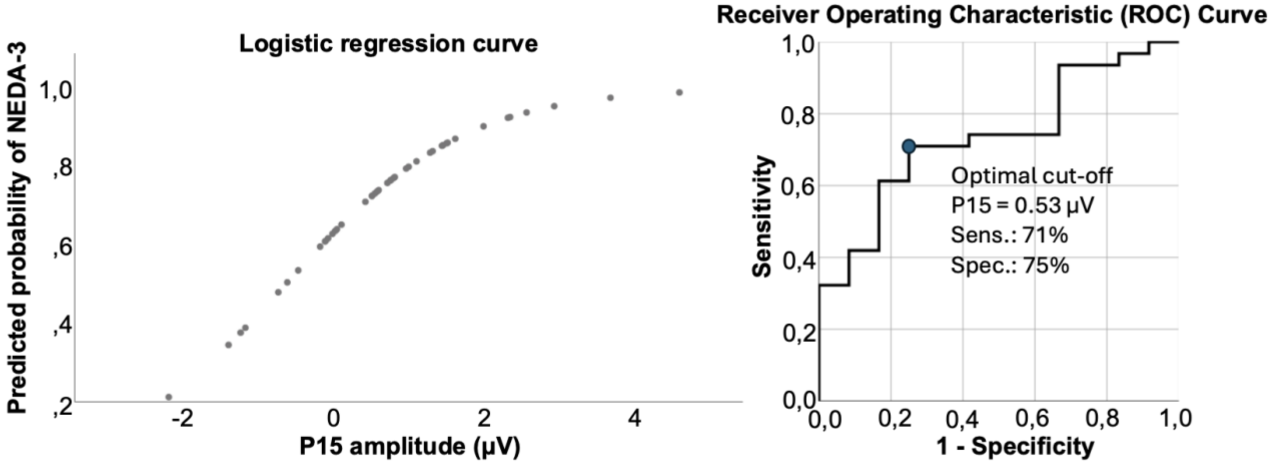

Supplementary Figure 4. Predictive value of P15 amplitude for NEDA-3 status at 2-year follow-up

(**A**) Logistic regression model showing the predicted probability of achieving No Evidence of Disease Activity (NEDA-3) as a function of P15 amplitude. Individual data points along the curve represent the estimated probability for each patient.

(**B**) Receiver Operating Characteristic (ROC) curve for the univariate logistic model based on P15 amplitude. The Area Under the Curve (AUC) was 0.737 (95% CI 0.581–0.892; p = 0.017).

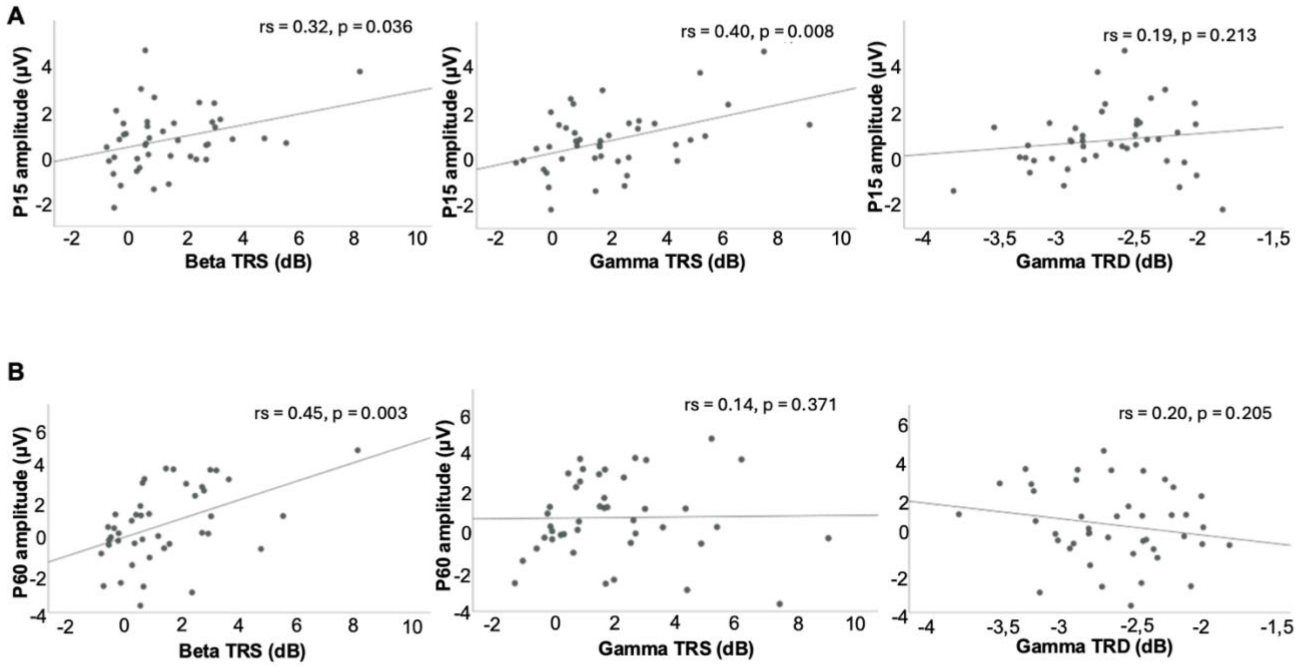

Supplementary Figure 5. Correlation between TEPs and TRSP in MS patients for features that differ from HCs

(A) Scatterplots showing the distribution of P15 amplitude values as a function of Beta band TMS-related synchronization (TRS) (left), Gamma TRS (center), and Gamma TMS-related desynchronization (TRD) (right).

(B) Scatterplots showing the distribution of P60 amplitude values as a function of Beta TRS (left), Gamma TRS (center), and Gamma TRD (right). Associations were assessed using Spearman's rank correlation coefficient (rs). Relevant statistics are reported in each panel.

Each dot represents an individual patient. The gray line indicates a linear fit provided for visualization purposes only. Spearman's ρ (rs) and corresponding p-values are reported in each panel.

TEP = TMS-evoked potentials; TRSP: TMS-related spectral perturbations; MS = multiple sclerosis; HCs: healthy controls.

Supplementary Table 1. Univariate Logistic regression results on NEDA-3 prediction

Variable	p-value	Exp(B)	95% CI Exp(B)
P15 amp.	**0.023**	2.321	1.120–4.810
P30 amp.	0.419	1.165	0.804–1.688
N45 amp.	0.520	1.164	0.733–1.847
P60 amp.	0.585	0.909	0.645–1.280
N100 amp.	0.718	0.964	0.792–1.174
P15 lat.	0.631	1.096	0.753-1.596
P30 lat.	0.731	1.058	0.766-1.462
N45 lat.	0.522	0.899	0.649-1.245
P60 lat.	0.478	1.076	0.879-1.318
N100 lat.	0.140	0.932	0.849-1.023
Beta TRS	0.302	1.261	0.812-1.958
Gamma TRS	0.591	1.090	0.795-1.496
Gamma TRD	0.423	0.527	0.110-2.259
